# Pathogen Evasion of Humoral Innate Immunity: Coping with C-Reactive Protein and Serum Amyloid A

**DOI:** 10.3390/ijms27021072

**Published:** 2026-01-21

**Authors:** Weichen Gong, Xuefei Cheng, Julio Villena, Haruki Kitazawa

**Affiliations:** 1 Laboratory of Animal Food Function, Graduate School of Agricultural Science, Tohoku University, Sendai 980-8577, Japan; jvillena@tohoku.ac.jp; 2 Livestock Immunology Unit, International Education and Research Center for Food and Agricultural Immunology (CFAI), Graduate School of Agricultural Science, Tohoku University, Sendai 980-8577, Japan; 3 Department of Microbiology and Immunology, Nihon University School of Dentistry at Matsudo, Chiba 271-8587, Japan; mase24010@g.nihon-u.ac.jp; 4 Laboratory of Respiratory Immunology (LaRI), Division of Animal Immunology and Omics, International Education and Research Center for Food and Agricultural Immunology (CFAI), Graduate School of Agricultural Science, Tohoku University, Sendai 980-8577, Japan

**Keywords:** C-reactive protein, serum amyloid A, humoral innate immune, pathogen evasion

## Abstract

C-reactive protein (CRP) and serum amyloid A (SAA) are classical acute-phase proteins that exemplify humoral innate immunity, the soluble arm of the host’s first-line defense. Beyond their traditional use as biomarkers of inflammation, both proteins function as active effectors against pathogens by binding microbial components, activating complements, and modulating inflammation. However, bacteria, viruses, and fungi have co-evolved diverse mechanisms to cope with or evade these host defenses. This review aims to summarize the current understanding of CRP and SAA as soluble innate immune effectors and to highlight pathogen strategies to counteract their antimicrobial pressure. We systematically surveyed and summarized evidence from experimental and clinical studies describing “function of CRP and SAA during infection”, “CRP and SAA in innate immune defense”, and “evasion mechanisms across bacterial, viral, and fungal pathogens”. CRP and SAA are rapidly upregulated in response to infection and contribute to pathogen recognition, opsonization, and inflammation. Pathogens, however, employ multiple coping strategies, including surface modification to block CRP binding, proteolytic degradation of acute-phase proteins, shielding within biofilms, and subversion of host signaling. These countermeasures enable microbes to reduce immune clearance and promote persistence. CRP and SAA represent central elements of humoral innate immunity, shaping the outcome of host–pathogen interactions. Pathogen adaptations to these proteins illustrate an ongoing evolutionary arms race between host defense and microbial survival. A deeper understanding of these processes may open avenues for novel therapeutic approaches, such as targeting microbial evasion factors or enhancing host acute-phase responses.

## 1. Introduction

The innate immune system provides the first line of defense against invading pathogens through both cellular and soluble components [1]. Among soluble factors, acute-phase proteins such as C-reactive protein (CRP) and serum amyloid A (SAA) play pivotal roles in humoral innate immunity. These proteins are rapidly induced in response to infection, tissue damage, or systemic inflammation and contribute to host defense by recognizing pathogen-associated molecular patterns, activating complement, facilitating opsonization, and modulating inflammatory signaling [2].

Beyond their immunological roles, CRP and SAA have gained significant attention as diagnostic and prognostic biomarkers of infection and inflammation in both human and veterinary medicine [3,4,5,6]. Owing to their rapid and robust induction during the acute-phase response, they serve as sensitive indicators of systemic inflammation, often rising within hours following pathogen invasion. CRP, in particular, is one of the most widely measured clinical biomarkers worldwide. In humans, elevated CRP levels are routinely used to distinguish bacterial from viral infections, to monitor treatment efficacy, and to predict clinical outcomes in sepsis, pneumonia, and other inflammatory diseases [3]. High-sensitivity CRP (hsCRP) assays have further extended its utility to cardiovascular risk assessment and chronic inflammatory disorders, reflecting low-grade systemic inflammation [7].

SAA has emerged as an equally important but less specific biomarker. It exhibits a faster and greater magnitude of increase than CRP—sometimes exceeding 1000-fold during acute inflammation—making it particularly useful for detecting early or subclinical infections [8]. In respiratory and gastrointestinal infections, SAA levels correlate strongly with disease severity and tissue damage [9,10]. Furthermore, because SAA is synthesized not only by the liver but also locally at sites of inflammation, its measurement may offer insights into localized immune activation that are not captured by systemic CRP levels [11]. Combined measurement of CRP and SAA has been shown to improve diagnostic precision, especially in distinguishing bacterial from non-bacterial inflammation and in assessing treatment responses [12].

In veterinary medicine, CRP and SAA have become essential tools for monitoring animal health and welfare. In livestock species such as cattle, pigs, and horses, SAA serves as a reliable indicator of infection, stress, and tissue injury, aiding in the early detection of respiratory or reproductive diseases [6]. In companion animals, including dogs and cats, CRP and SAA assays are increasingly incorporated into clinical diagnostics to guide antimicrobial therapy and assess postoperative recovery [4,5]. The conservation of their acute-phase kinetics across mammalian species underscores their evolutionarily conserved role in inflammation and host defense.

While CRP and SAA have long been used clinically as biomarkers of infection and inflammation, emerging evidence indicates that they are active participants in host defense rather than passive indicators [13]. They influence the outcome of bacterial, viral, and fungal infections, shaping immune responses and microbial survival. Simultaneously, pathogens have evolved strategies to resist, neutralize, or even exploit CRP and SAA, highlighting a coevolutionary interplay between host innate humoral defenses and microbial adaptation.

Although acute-phase proteins have been extensively discussed in the context of inflammation and infection, most existing reviews primarily emphasize their utility as clinical biomarkers or describe their functions within broader innate and adaptive immune networks. Far less attention has been paid to CRP and SAA as active, soluble effectors of humoral innate immunity, particularly from the perspective of host–pathogen interactions. Despite their importance, reviews focusing specifically on CRP and SAA as soluble innate effectors—and on the diverse pathogen strategies to overcome them—have not been published before.

Therefore, this review aims to synthesize current knowledge on: (i) the physiological roles of CRP and SAA during bacterial, viral, and fungal infections, and (ii) the mechanisms by which pathogens counteract or exploit these acute-phase proteins. To achieve this, we first provide a mechanistic overview of CRP- and SAA-mediated humoral innate immune functions, which establishes the selective pressures imposed on invading pathogens. We then integrate emerging evidence demonstrating how bacteria, viruses, and fungi have evolved distinct strategies to evade, neutralize, or repurpose these molecules to enhance survival and persistence.

Understanding these interactions may provide new insights into innate immune function, pathogen adaptation, and the development of host-directed therapeutic strategies.

## 2. Physiological Functions of SAA in Bacterial, Viral, and Fungal Infections

SAA is one of the most highly inducible acute-phase proteins and represents a key component of humoral innate immunity, the soluble defense system acting independently of immune cells [10,14]. During infection or tissue damage, hepatic expression of SAA increases dramatically under the stimulation of proinflammatory cytokines such as interleukin (IL)-1β, IL-6, and tumor necrosis factor (TNF)-α [15,16]. Although originally recognized as a precursor of amyloid A fibrils in chronic inflammation, SAA is now understood to function as an immunomodulatory and antimicrobial effector molecule that bridges innate recognition and inflammatory signaling [11,17,18].

In bacterial infections, SAA plays multiple roles in pathogen sensing and host protection. It binds to bacterial outer membrane components, including lipopolysaccharide (LPS) and outer membrane proteins, thereby contributing to pathogen recognition [19]. SAA can act as an opsonin, enhancing phagocytosis of *Escherichia coli* and *Staphylococcus aureus* by neutrophils and macrophages [20]. Furthermore, SAA activates Toll-like receptors (TLR2 and TLR4) and the formyl peptide receptor (FPR2), leading to chemotaxis and cytokine release that coordinate early inflammatory responses [21,22]. These activities collectively facilitate bacterial clearance, although excessive SAA-mediated inflammation may also contribute to tissue injury.

During viral infections, SAA expression is elevated in response to systemic cytokine release and interferon signaling [9,23]. Elevated SAA levels have been reported in patients infected with influenza virus, hepatitis B virus, and severe acute respiratory syndrome coronavirus 2 (SARS-CoV-2) [24,25]. Mechanistically, SAA can regulate the antiviral defense by modulating the balance between proinflammatory and regulatory cytokines. It may enhance recruitment of immune cells to infected tissues and restrict viral replication through indirect activation of TLR pathways [9]. Some evidence also suggests that SAA can interact with viral envelope components, although the functional significance of this interaction remains unclear [2,24,25].

In fungal infections, SAA contributes to antifungal immunity by promoting cytokine responses and modulating the differentiation of Th17 cells, which are essential for antifungal defense [26]. It can induce IL-17A and IL-22 production, supporting neutrophil activation and fungal clearance [27,28]. While direct binding between SAA and fungal cell wall polysaccharides such as β-glucans has been proposed, experimental confirmation is still limited [29].

**Figure 1:**
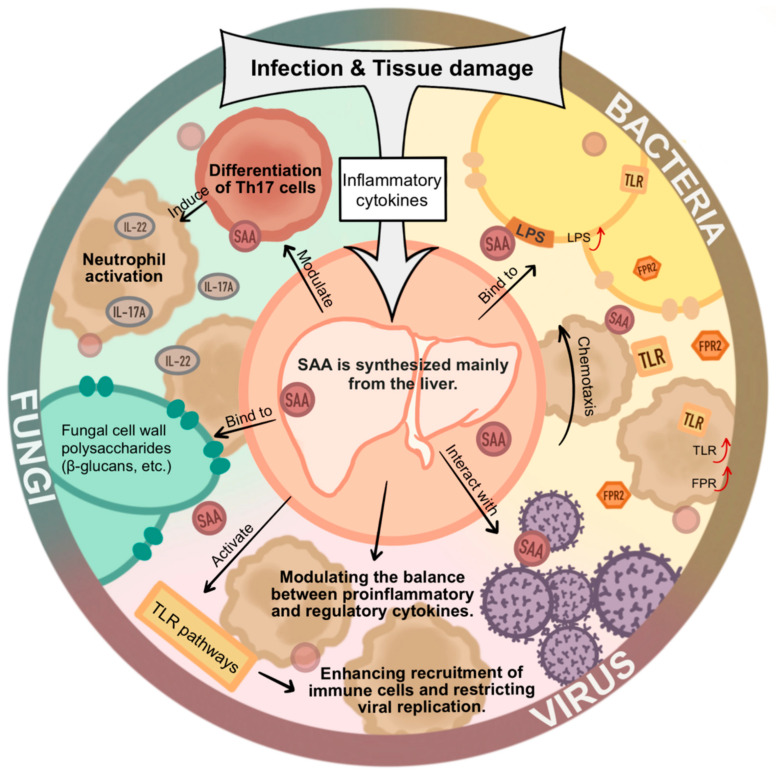
Physiological functions of SAA in bacterial, viral, and fungal infections. Upon infection, SAA is rapidly induced as a major acute-phase protein and functions as a key component of humoral innate immunity. SAA binds to microbial and host-derived ligands, modulates inflammatory signaling, promotes leukocyte chemotaxis and T cell differentiation, and regulates cytokine production. During bacterial infections, SAA influences complement activation, opsonization, and microbial stress adaptation. In viral infections, SAA shapes antiviral inflammation and tissue responses. In fungal infections, SAA contributes to immune recognition and modulation of antifungal immunity. Together, these functions position SAA as an active regulator of host–pathogen interactions across diverse microbial kingdoms (Some mechanisms depicted are based on indirect or emerging evidence, as discussed in the main text).

Collectively, these findings demonstrate that SAA is not merely an inflammatory biomarker but a multifunctional soluble immune effector that participates in pathogen recognition, immune cell recruitment, and inflammatory regulation across bacterial, viral, and fungal infections (Figure 1).

## 3. Physiological Functions of CRP in Bacterial, Viral, and Fungal Infections

CRP is a prototypical acute-phase reactant and one of the best-characterized molecules of humoral innate immunity [30]. Synthesized mainly in the liver under the control of IL-6 and other cytokines, CRP levels can increase more than a thousand-fold during systemic inflammation or infection [31]. As a soluble pattern-recognition molecule (PRM), CRP binds to pathogen- and damage-associated molecular patterns (PAMPs and DAMPs, respectively), facilitating complement activation and phagocytic clearance [32].

In bacterial infections, CRP exerts potent antibacterial activities through direct recognition and opsonization. The classic ligand of CRP is phosphocholine (PC), a structural motif present on the cell wall of many Gram-positive bacteria such as *Streptococcus pneumoniae* and *S. aureus*. Upon binding to PC residues, CRP triggers activation of the classical complement pathway via C1q recruitment, leading to opsonization and bacterial lysis [33,34]. This mechanism represents one of the most ancient and efficient humoral innate immune responses. CRP also enhances Fcγ receptor–mediated phagocytosis and modulates cytokine release by macrophages, thereby bridging innate recognition with cellular immune responses [35,36]. Interestingly, some bacteria, including *Haemophilus influenzae* and *Neisseria meningitidis*, modify their surface PC expression to evade CRP-mediated complement killing, underscoring the evolutionary interplay between CRP and bacterial pathogens [37,38].

In viral infections, the role of CRP is more complex. Elevated CRP levels are commonly observed in infections caused by influenza, hepatitis viruses, and SARS-CoV-2, where CRP reflects systemic inflammation rather than direct antiviral activity [39,40]. However, recent studies suggest that CRP may also participate in antiviral defense by modulating cytokine cascades and enhancing clearance of virus-infected apoptotic cells [41]. CRP can bind to exposed PC-like structures on infected or damaged host cells, marking them for removal by macrophages. Through this mechanism, CRP limits viral propagation and contributes to tissue homeostasis, although excessive CRP-mediated inflammation can exacerbate immunopathology, as observed in severe COVID-19 cases [34,42,43].

In fungal infections, CRP has been less extensively studied, but accumulating evidence indicates that it may contribute to antifungal immunity. CRP has been implicated in antifungal immune responses and can promote complement deposition on fungal surfaces [29]. While fungal cell wall polysaccharides such as glucans and mannans are potential ligands, direct molecular recognition by CRP has not been fully established. In infections caused by *Candida albicans* or *Aspergillus fumigatus*, CRP elevation correlates with disease activity and may participate in pathogen opsonization. Experimental models further suggest that CRP can enhance the phagocytic uptake of fungi by macrophages and neutrophils, acting as an opsonin similar to mannose-binding lectin (MBL) [44,45].

**Figure 2:**
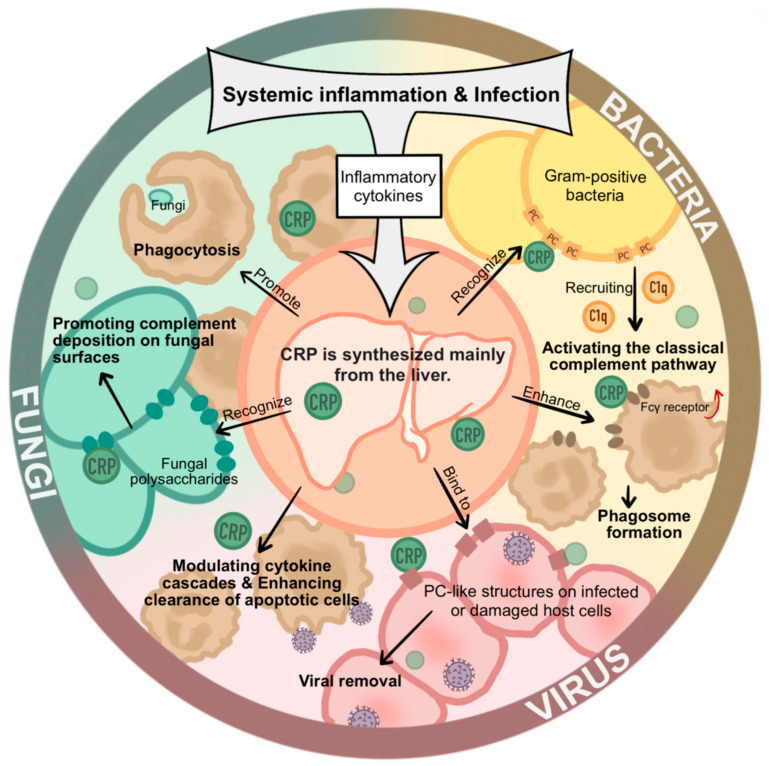
Physiological functions of CRP in bacterial, viral, and fungal infections. CRP acts as a soluble pattern-recognition molecule of humoral innate immunity by binding to pathogen-associated molecular patterns and damaged host components. CRP promotes complement activation, enhances opsonization, and facilitates pathogen clearance. In bacterial infections, CRP plays a prominent role in complement-mediated defense and inflammatory regulation. During viral and fungal infections, CRP modulates immune activation and contributes to host protection through complement-dependent and -independent mechanisms (Some mechanisms depicted are based on indirect or emerging evidence, as discussed in the main text).

Taken together, CRP serves as a multifunctional soluble immune effector that recognizes a wide range of microbial ligands, activates complement, and orchestrates inflammatory signaling. Its roles extend beyond a biomarker of infection to a central mediator of humoral innate defense against bacteria, viruses, and fungi (Figure 2). Consistent with this concept, recent studies have further expanded the known spectrum of CRP ligands, demonstrating its ability to bind capsular structures from at least 20 Gram-positive and Gram-negative bacterial pathogens. These findings underscore the remarkable versatility of CRP as a pattern-recognition molecule and support its broad involvement in host defense across diverse microbial species [46].

## 4. Shared Innate Immune Functions of CRP and SAA and the Convergent Pathogen Evasion Strategies

CRP and SAA are functionally analogous components of humoral innate immunity, acting as soluble PRMs that bridge innate recognition with effector mechanisms. Both are rapidly induced during the acute-phase response and bind to a wide range of microbial and host-derived ligands, including phosphocholine residues, lipopolysaccharides, and damaged membrane lipids [13]. First, both CRP and SAA are crucial in pattern recognition and opsonization. CRP recognizes microbial surface motifs such as phosphocholine-containing structures, capsular components, and altered host-derived ligands, whereas SAA interacts with bacterial outer membrane components, lipoproteins, and selected polysaccharides. Despite differences in ligand preference, both proteins can decorate microbial surfaces and enhance immune recognition, either directly or indirectly. Second, both CRP and SAA are also closely linked to complement activation. CRP activates the classical complement pathway through C1q binding, while SAA has been shown to modulate complement activity and amplify inflammatory cascades in a context-dependent manner. Through these mechanisms, CRP and SAA promote complement deposition, opsonophagocytosis, and—in some settings—direct microbial killing. Third, CRP and SAA exert broad immunomodulatory effects by shaping cytokine production and leukocyte responses. Both proteins can influence neutrophil recruitment, macrophage activation, and the balance between pro-inflammatory and regulatory cytokines, thereby fine-tuning host responses to infection. These effects are particularly relevant at mucosal surfaces and early infection sites, where rapid soluble immune responses precede cellular immunity.

Because of their overlapping biological functions, many pathogens have evolved convergent strategies to counteract the pressures imposed by CRP and SAA. These include (i) surface sequestration or binding of CRP and SAA by bacterial or fungal outer membrane and cell wall components to prevent complement activation; (ii) proteolytic degradation of acute-phase proteins by secreted microbial enzymes; (iii) biofilm formation that limits access of soluble immune factors; and (iv) molecular mimicry or modulation of host inflammatory pathways to suppress their induction [47,48,49,50,51,52,53,54,55,56,57,58,59,60,61,62,63,64]. Viruses, lacking metabolic activity, tend to exploit CRP- and SAA-mediated inflammation indirectly—by enhancing viral entry or persistence within inflamed tissues—yet the underlying principle remains the same: attenuating or hijacking humoral innate defenses to ensure survival and propagation [41,65,66,67,68,69,70,71,72].

Thus, despite their distinct structural origins, CRP and SAA represent parallel arms of the soluble innate immune network, and the pathogens’ responses to them reveal shared evolutionary pressures. Studying these common evasion pathways not only clarifies how microbes adapt to host immunity but also provides a conceptual framework for designing therapeutic interventions that reinforce the humoral innate barrier against diverse infectious agents.

## 5. Bacterial Strategies to Cope with Pressure of CRP and SAA

**Figure 3:**
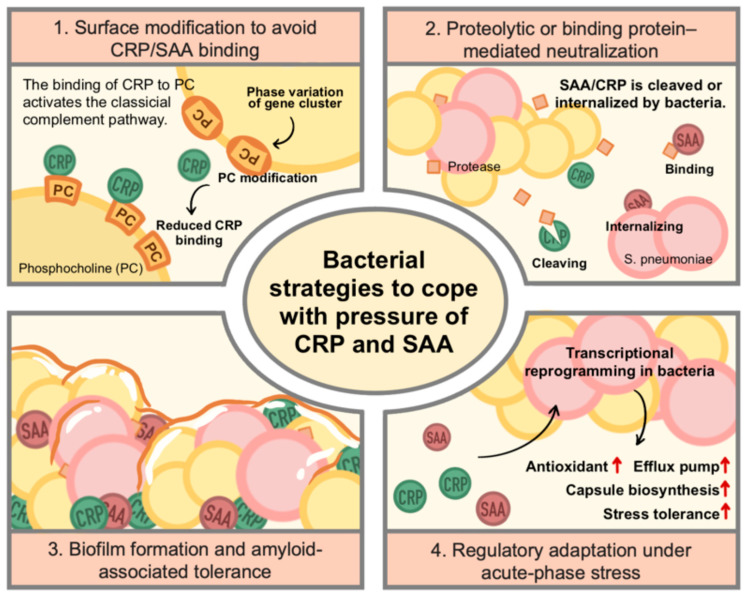
Bacterial strategies to cope with CRP- and SAA-mediated immune pressure. Bacteria have evolved multiple strategies to counteract the antimicrobial and immunomodulatory functions of the acute-phase proteins CRP and SAA. These strategies include (1) surface modification, such as alteration or masking of outer membrane and cell wall components to reduce CRP and SAA binding; (2) neutralization, achieved through proteolytic cleavage of CRP or SAA or by bacterial surface proteins that sequester and functionally inactivate these host factors; (3) biofilm-mediated resistance, in which CRP and SAA are incorporated into the biofilm matrix, enhancing biofilm stability while limiting their immune effector functions; and (4) genetic and metabolic adaptation, whereby bacteria modulate gene expression to improve tolerance to immune-mediated stress. Together, these mechanisms enable bacterial survival and persistence under humoral innate immune pressure (Some mechanisms depicted are based on indirect or emerging evidence, as discussed in the main text).

Among all types of pathogens, the interactions between bacteria and acute-phase proteins such as CRP and SAA have been the earliest discovered and most extensively characterized. Since both proteins were initially identified as markers of bacterial infection, much of our understanding of their immunological roles originates from bacterial models. Studies across *Streptococcus*, *Staphylococcus*, *Pseudomonas*, and *Enterobacteriaceae* have revealed that CRP and SAA exert potent antibacterial effects through complement activation, opsonization, and modulation of inflammatory signaling. In response, bacteria have evolved diverse mechanisms to resist, evade, or even exploit these host factors. The following section summarizes the major bacterial strategies to cope with CRP- and SAA-mediated immune pressures, highlighting their molecular and physiological diversity (Figure 3).

### 5.1. Surface Modification to Avoid CRP/SAA Binding

A classic example is *S. pneumoniae*, the original target of CRP recognition. CRP binds to PC residues on pneumococcal teichoic acids, activating the classical complement pathway [47]. However, *S. pneumoniae* can alter PC expression through phase variation in the licD gene cluster, reducing CRP binding and complement sensitivity [48]. Similarly, *H. influenzae* and *N. meningitidis* downregulate PC or modify their outer membrane structures, enabling them to escape CRP-mediated opsonization [49]. Some Gram-negative bacteria also modify lipid A acylation or surface charge, indirectly affecting CRP interaction and complement deposition [50].

### 5.2. Proteolytic or Binding Protein–Mediated Neutralization

Certain bacteria secrete proteases capable of degrading host acute-phase proteins. For instance, *Pseudomonas aeruginosa* elastase can cleave CRP, thereby diminishing its ability to activate complement and promote opsonization [51]. Other bacteria produce surface-binding proteins that hijack CRP or SAA and prevent their immune functions. *S. aureus* secretes protein A and Sbi, which classically bind the Fc region of immunoglobulins but can also interact with CRP, thereby disrupting complement activation [52,53]. In *Enterobacteriaceae* species, outer membrane proteins can bind SAA, potentially neutralizing its antimicrobial effect or facilitating bacterial adhesion to host tissues [20,54]. Interestingly, certain bacteria do not merely evade but actively exploit acute-phase proteins. A recent study demonstrated that *S. pneumoniae* can internalize the host protein SAA1 to enhance survival under acidic stress. This finding highlights an unexpected facet of bacterial survival: instead of merely resisting host defense proteins, some pathogens can repurpose them as functional shields under environmental stress [55,56].

### 5.3. Biofilm Formation and Amyloid-Associated Tolerance

Both CRP and SAA have been detected within bacterial biofilms, where they may modulate bacterial aggregation and immune evasion [57]. SAA, due to its amyloidogenic nature, can interact with bacterial amyloid proteins such as curli and FapC, potentially reinforcing biofilm stability [58,59]. In response, bacteria exploit biofilm-associated matrices to shield themselves from CRP- and complement-mediated killing [60]. This strategy not only provides physical protection but also creates a microenvironment where acute-phase proteins are sequestered and inactivated. For example, *Klebsiella pneumoniae* and *E. coli* biofilms show increased resistance to CRP-dependent complement lysis compared with their planktonic counterparts [61,62,63].

### 5.4. Regulatory Adaptation Under Acute-Phase Stress

Exposure to CRP or SAA can induce transcriptional reprogramming in bacteria, enhancing stress tolerance. Transcriptomic studies show that *Salmonella enterica* exposed to SAA upregulates antioxidant and efflux pump genes, potentially as a countermeasure to host-derived reactive oxygen species and lipid peroxidation [64]. Similarly, *S. aureus* under CRP stress upregulates capsule biosynthesis genes, providing additional protection against opsonization [65].

Collectively, these findings reveal that bacteria actively adapt to the presence of CRP and SAA through structural, enzymatic, and biofilm-based strategies, highlighting the coevolution between host humoral immunity and microbial survival mechanisms.

## 6. Viruses Employ Distinct Mechanisms to Escape CRP and SAA Surveillance

**Figure 4:**
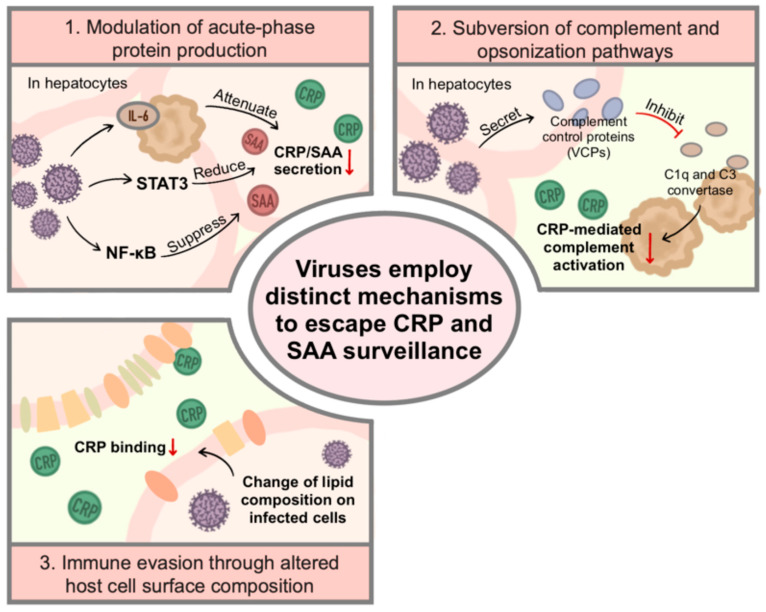
Viral strategies to evade CRP- and SAA-mediated humoral innate immunity. Viruses employ multiple mechanisms to counteract the antiviral functions of the acute-phase proteins CRP and SAA. These strategies include (1) suppression of acute-phase protein production, whereby viral infection modulates signaling pathways in hepatocytes to reduce CRP and SAA synthesis and secretion; (2) inhibition of complement activation, achieved through viral-encoded complement control proteins that interfere with CRP- and SAA-dependent complement pathways; and (3) alteration of host cell membrane composition, in which viruses remodel lipid and protein components of infected cell membranes, thereby limiting CRP binding and impairing its effector functions. Collectively, these mechanisms enable viruses to dampen humoral innate immune responses and promote viral persistence and spread (Some mechanisms depicted are based on indirect or emerging evidence, as discussed in the main text).

Unlike bacteria, viruses lack their own metabolic or structural complexity, yet they have evolved intricate ways to manipulate and evade humoral innate immunity. Also, viruses predominantly evade CRP- and SAA-mediated humoral innate immunity through indirect, host-dependent mechanisms, reflecting their obligate intracellular lifestyle. During viral infection, host acute-phase proteins such as CRP and SAA are rapidly upregulated as part of the systemic inflammatory response [57]. Although these proteins can contribute to antiviral defense by modulating cytokine cascades, recruiting immune cells, and promoting the clearance of apoptotic cells, many viruses have developed distinct molecular strategies to avoid, suppress, or exploit CRP- and SAA-mediated immune functions (Figure 4).

### 6.1. Modulation of Acute-Phase Protein Production

A common viral evasion strategy involves the suppression or dysregulation of acute-phase protein synthesis. Certain hepatotropic viruses, such as hepatitis B virus (HBV) and hepatitis C virus (HCV), can directly interfere with IL-6 signaling pathways in hepatocytes, thereby attenuating CRP transcription [66,67,68]. HBV X protein (HBx) has been shown to downregulate STAT3 activation, leading to reduced CRP production during the early infection phase [69]. Similarly, HCV core and non-structural 5A proteins (NS5A protein) modulate NF-κB activity to suppress SAA expression [70]. This suppression may limit early antiviral signaling and complement activation, providing the virus a temporal advantage to establish infection before systemic inflammation peaks.

### 6.2. Subversion of Complement and Opsonization Pathways

CRP can recognize PC-like structures on viral envelopes or infected host cell membranes, marking them for complement activation [41]. However, many viruses encode complement-regulatory proteins or recruit host complement inhibitors to their surface. For instance, vaccinia virus and other poxviruses express complement control proteins (VCPs) that inhibit C1q and C3 convertase, effectively blocking CRP-mediated complement activation [71,72]. Influenza virus incorporates host-derived CD55 and CD59 into its envelope, which prevents membrane attack complex formation despite CRP deposition [73]. These mechanisms neutralize one of CRP’s major effector functions—classical pathway activation—and allow viruses to persist in the extracellular milieu.

### 6.3. Immune Evasion Through Altered Host Cell Surface Composition

Some viruses manipulate the lipid composition of infected cells to reduce CRP recognition [74]. For example, influenza virus and certain herpesviruses alter membrane phospholipid content, decreasing accessible PC residues and thus limiting CRP binding [75]. This strategy minimizes opsonization and complement-mediated clearance of infected cells while preserving viral replication niches.

Collectively, these findings indicate that viruses interact with CRP and SAA in a highly context-dependent manner. Some suppress acute-phase protein synthesis to evade early recognition, others neutralize their effector functions through complement regulation, and still others exploit their inflammatory potential to promote immune dysregulation. These multifaceted viral strategies underscore the evolutionary tension between host humoral innate defenses and viral immune evasion, highlighting CRP and SAA as both sentinels and potential liabilities within the antiviral response network.

## 7. Fungi Adapt to CRP and SAA Pressure Through Cell Wall Remodeling and Immune Modulation

**Figure 5:**
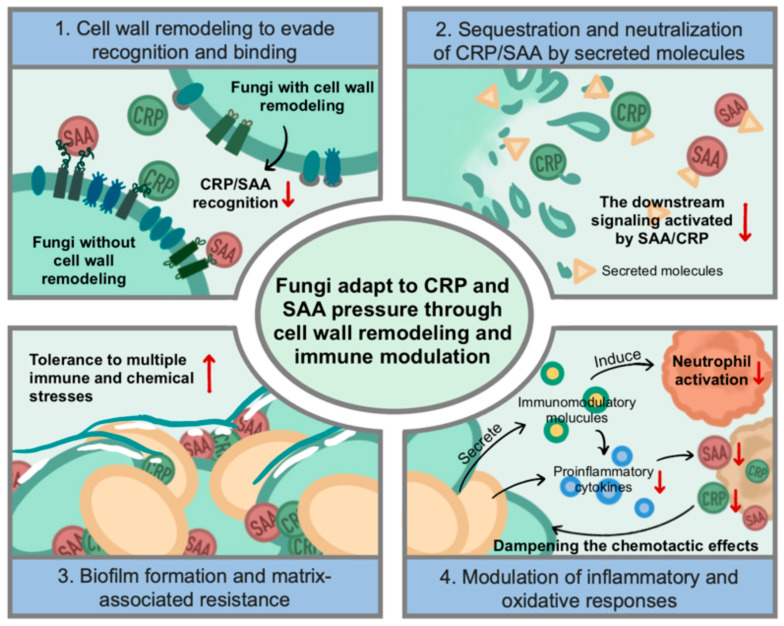
Fungal strategies to counteract SAA- and CRP-mediated humoral innate immune pressure. Pathogenic fungi employ diverse mechanisms to evade or suppress the immune functions of the acute-phase proteins SAA and CRP. These strategies include (1) cell wall remodeling, whereby alterations in polysaccharide composition and surface architecture limit recognition and binding by SAA and CRP; (2) secretion of extracellular factors, which interfere with downstream immune signaling pathways and prevent activation of complement and inflammatory responses; (3) biofilm-associated sequestration, in which SAA and CRP are incorporated into the fungal biofilm matrix, enhancing biofilm stability while reducing their antimicrobial activity; and (4) immune modulation, achieved through fungal-derived immunoregulatory molecules that suppress neutrophil activation and downregulate SAA and CRP expression. Together, these mechanisms facilitate fungal persistence under humoral innate immune pressure (Some mechanisms depicted are based on indirect or emerging evidence, as discussed in the main text).

Among eukaryotic pathogens, fungi represent a unique challenge for the host immune system because of their complex cell wall composition and morphological plasticity. CRP and SAA, as soluble mediators of humoral innate immunity, play emerging roles in antifungal defense by promoting opsonization, complement activation, and cytokine modulation. However, pathogenic fungi such as *C. albicans* and *A. fumigatus*, have evolved multiple adaptive mechanisms to tolerate or counteract the pressure imposed by these acute-phase proteins (Figure 5).

### 7.1. Cell Wall Remodeling to Evade Recognition and Binding

The fungal cell wall is a dynamic structure composed of β-glucans, mannans, and chitin, which serve as key targets for host recognition [76,77]. CRP has been shown to bind to fungal polysaccharides, particularly glucans, facilitating complement activation and immune cell recruitment [78,79]. To avoid this, many fungi remodel their cell wall during infection. *C. albicans* can mask β-glucans with an outer mannan layer, thereby reducing CRP and SAA binding [80]. Under host-like conditions, *A. fumigatus* conidia conceal immunogenic polysaccharides beneath a rodlet and melanin layer, preventing acute-phase proteins from accessing their targets [81]. *Cryptococcus neoformans* employs a thick polysaccharide capsule composed of glucuronoxylomannan (GXM), which is well known to impair complement activation and neutrophil-mediated clearance [82]. Although direct inhibition of CRP binding by GXM has not been experimentally demonstrated, capsule-mediated shielding may limit access of soluble innate immune factors, including CRP.

### 7.2. Sequestration and Neutralization of CRP/SAA by Secreted Molecules

Some fungi secrete extracellular polysaccharides or proteins that act as decoys for acute-phase proteins. The soluble GXM released by *C. neoformans* can bind CRP and complement components in the extracellular space, diverting opsonization away from the fungal surface [82]. Similarly, *C. albicans* secretes aspartyl proteases (SAP family) and other hydrolytic enzymes that may degrade or modify bound host proteins, although their direct activity against CRP or SAA has not been fully confirmed [83,84]. These mechanisms collectively reduce effective concentrations of functional acute-phase proteins near fungal cells, thereby weakening host defense.

### 7.3. Biofilm Formation and Matrix-Associated Resistance

Biofilm growth is a critical adaptive strategy that confers tolerance to multiple immune and chemical stresses. CRP and SAA can penetrate fungal biofilms but are often trapped within the extracellular matrix, where their immune functions are blunted [85]. *C. albicans* biofilms, rich in β-1,3-glucan and mannan, exhibit marked resistance to complement activation and opsonization, suggesting that biofilm matrix components physically and functionally sequester acute-phase proteins [86,87]. Moreover, SAA has been detected within fungal biofilms, where it may paradoxically enhance matrix stability by cross-linking extracellular proteins, indirectly supporting fungal persistence [26].

### 7.4. Modulation of Inflammatory and Oxidative Responses

SAA can activate host cells to release IL-17A, IL-22, and TNF-α, key cytokines in antifungal immunity [88]. To counteract this, fungi deploy immunomodulatory molecules that suppress or redirect host responses. *A. fumigatus* secretes galactosaminogalactan (GAG), which inhibits neutrophil activation and reduces cytokine release triggered by SAA [89,90]. Likewise, *C. neoformans* polysaccharides attenuate the production of proinflammatory cytokines, dampening the chemotactic effects of both CRP and SAA [91]. By modulating host signaling cascades, fungi limit the proinflammatory amplification that these acute-phase proteins normally induce.

Collectively, these findings reveal that fungi employ a combination of physical shielding, enzymatic neutralization, and immune modulation to withstand CRP- and SAA-mediated pressures. Unlike bacteria, which often degrade or internalize these proteins, fungi primarily rely on structural remodeling and immune evasion through biofilm and capsule formation. This adaptive interplay underscores the importance of humoral innate factors in shaping fungal pathogenesis and suggests that CRP and SAA not only serve as biomarkers of infection but also actively influence fungal survival and immune outcomes.

## 8. Conclusions and Perspectives

CRP and SAA, as key mediators of humoral innate immunity, play a crucial role in the interface between host defense and pathogen survival. They act as early sentinels, recognizing microbial and damage-associated patterns, activating complement, promoting opsonization, and orchestrating inflammatory responses. Across bacteria, viruses, and fungi, these proteins not only serve as biomarkers of infection but also exert direct antimicrobial and immunomodulatory functions [2]. Given the widespread clinical use of CRP and SAA as biomarkers of infection and inflammation, pathogen strategies that modulate or evade these acute-phase proteins may have important diagnostic implications. In bacterial infections, robust induction of CRP and SAA is often interpreted as a hallmark of acute inflammation; however, pathogens that degrade, sequester, or internalize these proteins may attenuate their measurable levels or alter their functional availability at infection sites. Such mechanisms could contribute to discordant clinical presentations in which inflammatory burden is underestimated despite ongoing infection. In viral infections, CRP and SAA levels are typically lower than in bacterial disease, a distinction frequently used in clinical decision-making. Nevertheless, viral suppression of hepatic acute-phase protein synthesis or indirect interference with CRP- and SAA-associated complement activity may further blur this distinction, particularly in severe or chronic viral infections. Similarly, in invasive fungal diseases, muted or atypical CRP/SAA responses have been reported in some clinical contexts, raising the possibility that fungal immune evasion strategies contribute to misleading biomarker profiles.

These observations suggest that CRP and SAA should not be interpreted solely as passive indicators of inflammation, but rather as dynamic components of host–pathogen interaction. A deeper understanding of how pathogens actively modulate CRP and SAA may improve diagnostic accuracy and support the development of more nuanced biomarker-based algorithms that integrate pathogen type, disease stage, and immune evasion mechanisms.

Pathogens, in turn, have evolved diverse strategies to counteract CRP and SAA. Bacteria deploy proteases, surface-binding proteins, and biofilm-associated defenses, and some species, such as *S. pneumoniae*, actively internalize SAA to enhance environmental tolerance [55,56]. At present, this mechanism has been experimentally demonstrated only in *S. pneumoniae*, and its prevalence across different pneumococcal lineages or other bacterial species remains unknown. Nevertheless, this finding raises the intriguing possibility that certain bacteria may actively repurpose host acute-phase proteins as functional stress-adaptation factors rather than merely neutralizing them. Future studies combining comparative genomics, targeted mutagenesis, and in vivo infection models will be required to determine whether SAA internalization represents a broader bacterial strategy or a species-specific adaptation. Viruses manipulate acute-phase protein production, subvert complement pathways, and exploit SAA-mediated inflammation to their advantage [70]. Fungi rely on cell wall remodeling, extracellular decoys, biofilm entrapment, and immunomodulatory molecules to evade or neutralize CRP and SAA activity [82]. Collectively, these mechanisms highlight an evolutionary arms race, where soluble innate immune proteins and pathogen evasion strategies coevolve.

While this review focuses on CRP and SAA as soluble effectors of humoral innate immunity during bacterial, viral, and fungal infections, several related areas were intentionally not covered. Parasitic infections were excluded because evidence linking parasite-derived ligands to direct CRP- or SAA-mediated recognition remains limited and mechanistically distinct from microbial systems discussed here. Interactions between CRP/SAA and the commensal microbiota were also beyond the scope of this review, as these relationships are often context-dependent and intertwined with metabolic and chronic inflammatory processes rather than acute infection. In addition, chronic inflammatory and autoimmune conditions were not discussed in detail, despite their strong association with sustained CRP and SAA elevation, because the focus of this article is on acute host–pathogen interactions and immune evasion strategies. These omissions represent important directions for future investigation and highlight the need for dedicated reviews addressing CRP and SAA functions in non-infectious or host–microbiota contexts.

It is also important to note that a substantial proportion of the mechanistic insights discussed in this review are derived from murine models and in vitro cell culture systems, and that significant species-specific differences exist in both CRP and SAA biology. While CRP is highly inducible in humans during acute inflammation, murine CRP displays a more modest acute-phase response, and its contribution to systemic immunity may differ quantitatively and qualitatively between species. In contrast, SAA exhibits pronounced heterogeneity across mammals, with marked differences in isoform composition, tissue distribution, and inflammatory functions.

Looking forward, several avenues warrant further investigation. First, the precise molecular interactions between CRP/SAA and microbial ligands remain incompletely characterized, particularly for viruses and fungi. Second, understanding how pathogens repurpose or hijack acute-phase proteins may uncover novel targets for therapeutic intervention. Finally, integrating CRP and SAA measurements with host–pathogen modeling could improve infection diagnostics and inform strategies to enhance innate humoral immunity.

A critical yet underexplored challenge in the field is to disentangle the relationship between local and systemic concentrations of CRP and SAA during infection. While circulating levels of these acute-phase proteins are routinely measured, far less is known about how their local abundance at infection sites correlates with systemic readouts, or how these dynamics evolve over time. Addressing this gap is essential for the rational interpretation of CRP and SAA as biomarkers, particularly because tissue injury or invasive sampling procedures themselves can artificially elevate acute-phase protein production. Improved understanding of local–systemic CRP and SAA coupling may enable the development of minimally invasive or non-invasive monitoring strategies that allow longitudinal tracking of acute-phase protein dynamics without confounding inflammatory stimuli. Continuous or repeated measurements could provide valuable insight into how CRP and SAA trajectories reflect distinct stages of bacterial, viral, or fungal infection, disease progression, and resolution. In parallel, experimental systems that permit independent and controlled modulation of CRP or SAA levels are urgently needed. The use of cell-based models or animal systems in which CRP or SAA expression can be selectively induced—without broadly activating inflammatory pathways—would allow direct assessment of their individual contributions to host defense and immune regulation. Such approaches would help disentangle the intrinsic effector functions of CRP and SAA from secondary inflammatory effects and provide a clearer framework for evaluating their therapeutic potential. Taken together, these strategies may transform CRP and SAA from static biomarkers into dynamic indicators and manipulable components of humoral innate immunity.

From a therapeutic perspective, modulating CRP and SAA expression or their downstream signaling pathways represents a promising but largely underexplored strategy for enhancing host resistance to infection. Given their roles as soluble PRMs that bridge innate sensing with effector activation, controlled induction of these acute-phase proteins could potentially strengthen early immune responses [92]. Pharmacological agents or immunomodulatory adjuvants that activate hepatic acute-phase synthesis—such as IL-6 agonists, TLR ligands, or small-molecule inducers of STAT3 signaling—might be harnessed to transiently elevate CRP and SAA levels during the initial phase of infection. Such approaches could amplify complement activation, enhance opsonization, and promote the recruitment of neutrophils and macrophages to infection sites.

Alternatively, direct activation of downstream effector pathways may provide therapeutic benefits without necessitating systemic elevation of acute-phase proteins. For instance, agents that mimic CRP or SAA binding to Fcγ receptors or scavenger receptors could stimulate phagocytosis, oxidative burst, and neutrophil extracellular trap (NET) formation, thereby accelerating pathogen clearance. Similarly, targeted delivery of recombinant CRP or SAA, or their bioactive fragments, could be explored as immunostimulatory therapeutics, particularly in immunocompromised hosts where the acute-phase response is blunted. Experimental studies have already shown that recombinant CRP enhances bacterial clearance and reduces lethality in murine sepsis models, while SAA analogs modulate leukocyte chemotaxis and cytokine balance [93].

Additionally, the timing and magnitude of CRP- and SAA-mediated responses are likely to be critical determinants of their beneficial versus detrimental effects during infection. In the early phase of infection, rapid induction of CRP and SAA is generally advantageous, as these acute-phase proteins facilitate prompt activation of complement, enhance opsonization, and accelerate downstream innate immune responses. Augmenting CRP or SAA availability during this early window may therefore represent a potential strategy to boost host defense and limit initial pathogen expansion. However, sustained elevation of CRP and SAA during prolonged or unresolved infection may have unintended consequences. Accumulating evidence suggests that chronic exposure to high levels of acute-phase proteins can be exploited by certain bacteria and fungi to reinforce biofilm formation, promote persistence, and reduce susceptibility to antimicrobial treatment. In this context, prolonged CRP and SAA signaling may shift from being protective to permissive, facilitating microbial adaptation rather than clearance. These observations support a stage-dependent, bidirectional therapeutic model in which CRP and SAA are selectively enhanced during early infection but actively downregulated during later disease stages if levels remain persistently high. Therapeutic strategies aimed at reducing excessive acute-phase protein signaling—either by limiting CRP/SAA production or by modulating their downstream pathways—may disrupt biofilm stability and restore antimicrobial efficacy. Such approaches could be particularly valuable when combined with antibiotics or antifungals, as weakening biofilm-associated tolerance may enhance drug penetration and microbial killing.

Overall, these potential strategies underscore the therapeutic promise of harnessing the humoral arm of innate immunity as an adjunct to conventional antimicrobial approaches. However, the challenge lies in achieving a fine balance—amplifying beneficial immune activation while avoiding excessive inflammation or tissue injury. Future research should therefore aim to delineate the optimal timing, dosage, and molecular pathways through which CRP and SAA can be safely leveraged for host-directed therapies.

In conclusion, CRP and SAA function not only as indicators of inflammation but also as active modulators of pathogen clearance and environmental adaptation. The dynamic interplay between these proteins and pathogens underscores the complexity of host–pathogen interactions and provides a framework for future research on humoral innate immunity and microbial survival strategies.

## Data Availability

No new data were created or analyzed in this study. Data sharing is not applicable to this article.
